# Epidermal Growth Factor Receptor Mutation in Resectable Lung Cancer: Association With Survival Outcomes

**DOI:** 10.1016/j.atssr.2025.09.007

**Published:** 2025-10-06

**Authors:** Charles E. Bardawil, Kathryn Demanelis, Yota Suzuki, Summer Mazur, Hannah Udoh, Adam Soloff, Rajeev Dhupar

**Affiliations:** 1 Surgical and Research Services, VA Pittsburgh Healthcare System, Pittsburgh, Pennsylvania; 2 Department of Cardiothoracic Surgery, University of Pittsburgh School of Medicine, Pittsburgh, Pennsylvania; 3 Department of Medicine, University of Pittsburgh School of Medicine, Pittsburgh, Pennsylvania; 4 Department of Cardiothoracic Surgery, Wake Forest University Health Sciences, Winston-Salem, North Carolina

## Abstract

**Background:**

Case series suggest comparable long-term survival between patients with epidermal growth factor receptor–mutant (EGFRmu) and wild-type (EGFRwt) lung cancer after surgery. We aimed to analyze our experience with stage I-III adenocarcinoma patients who underwent surgery, hypothesizing similar long-term survival.

**Methods:**

From 2733 lobectomies (2013-2023), 578 adenocarcinoma patients underwent complete mutational analysis. Exclusions were stage IV disease and inadequate EGFR testing. Of 112 EGFRmu and 466 EGFRwt patients, baseline characteristics were compared, and survival trends and prognostic factors were analyzed.

**Results:**

EGFRmu patients were more often female (*P* < .001), never-smokers (*P* < .001), and had stage I disease (*P* < .001). EGFRmu patients had better 5- and 10-year survival (55% vs 25%, *P* < .001), which may reflect baseline differences. Multivariable analysis identified tumor stage, age, male sex, and EGFRwt as predictors of mortality. Three-year disease-free survival was higher in EGFRmu patients compared with those with EGFRwt (75% vs 58%, *P* = .003). In multivariable analysis, EGFRwt status was not predictive of 5-year mortality in the absence of metastatic recurrence (hazard ratio, 1.05; 95% CI, 0.44-2.51; *P* = .92), but was significantly associated with increased risk of death following metastatic recurrence (hazard ratio, 1.77; 95% CI, 1.01-3.11; *P* = .048).

**Conclusions:**

EGFRmu patients have distinct clinical characteristics and potentially better long-term survival, suggesting a need for tailored strategies in screening, research, and treatment.

## Visual Abstract

**Figure:**
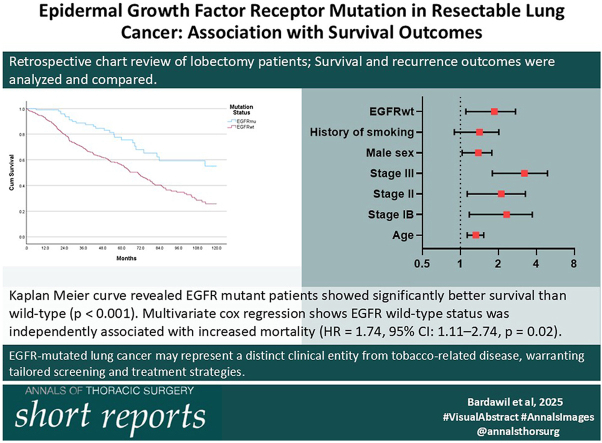

**In Short**

▪ Patients with epidermal growth factor receptor–mutant lung cancer showed significantly better 5- and 10-year survival than wild-type.
▪ Epidermal growth factor receptor wild-type status confers worse survival after disease recurrence compared with epidermal growth factor receptor–mutant lung cancer.
▪ Caution should be taken as epidermal growth factor receptor wild-type and mutant patients are highly different at baseline, which influence survival outcomes.

Patients with non-small cell lung cancer (NSCLC) harboring epidermal growth factor receptor (EGFR) mutations can benefit from tyrosine kinase inhibitors (TKIs), showing clinical utility in both surgical and nonsurgical settings.^1^^,^^2^ EGFR mutations are more common in never-smokers, female individuals, and Asian individuals.^3^ While tobacco smoke remains the most well-established risk factor for lung cancer, EGFR-mutant (EGFRmu) and EGFR wild-type (EGFRwt) tumors arise in populations with markedly different risk factors and demographics. These distinctions suggest that the underlying pathobiology may also differ, raising the possibility that long-term outcomes could vary between these groups. Assessing such differences could inform more tailored strategies for prevention, treatment, and prognostication. Recent case series^4^^,^^5^ suggested similar long-term survival between EGFRmu and EGFRwt patients undergoing curative-intent surgery, often including TKI therapy. However, due to a limited number of EGFRmu patients (50 and 238) with 5-year follow-up, we aimed to expand upon these findings. We hypothesized that EGFRmu and EGFRwt patients differ in their baseline characteristics and thus survival outcomes; however, when adjusting for potential confounders in multivariable analysis, their survival outcomes appear similar.

## Patients and Methods

Lung adenocarcinoma patients who underwent lobectomy between January 2013 and December 2023 at the University of Pittsburgh Medical Center were retrospectively reviewed using a prospectively collected surgical database. Only those with available mutational analysis were included (Supplemental Figure 1). Patients who did not have EGFR testing performed and had evidence of recurrence prior to surgical treatment were excluded. Only lobectomies were used, as this approach represented both the curative-intent standard during the study period and allowed for longer-term follow-up, enabling robust survival analysis. This extended follow-up was facilitated by consistent data capture within a maintained institutional database. Clinical, demographic, and pathological data were included. The study was approved by the institutional review board (STUDY23100047; December 15, 2023), and informed consent was waived due to its retrospective design.

Baseline characteristics of patients with EGFRmu and EGFRwt tumors were compared using the χ^2^ test for categorical variables and independent sample 2-sided *t* tests for continuous variables. Survival analysis evaluated 5-year and 10-year survival outcomes and 3-year disease-free survival. Kaplan-Meier estimation generated survival curves, with differences assessed using the log-rank test. Multivariable Cox regression estimated hazard ratios (HRs) and 95% CIs, incorporating EGFR status while adjusting for age, tumor stage (stage IA as reference category), sex, and smoking status, known predictors of mortality in NSCLC. Statistical analysis was performed using IBM SPSS statistics, version 27.0 (IBM Corp), with *P* values < .05 considered statistically significant.

## Results

### Cohort Characteristics

From 2013 to 2023, a total of 2733 lobectomies were performed, with 1612 adenocarcinomas. Post exclusion, 578 patients were included: 112 EGFRmu and 466 EGFRwt. The cohort was predominantly female (56%), had smoking history (81%), and exhibited heterogeneous pathologic stage distribution (Table 1). EGFRmu were more likely to be female (79% vs 50%, *P* < .001), never-smokers (49% vs 12%, *P* < .001), and have lower pathologic stage (52% vs 32% in stage I, *P* < .001; Table 2) than EGFRwt. Additionally, EGFRmu had smaller tumor size (2.6 vs 3.0 cm, *P* = .01) and were more likely to present with synchronous tumors (11% vs 1%, *P* < .001).

**Table 1:** Baseline Cohort Characteristics

Characteristics (N = 578)	Value
Mutation status	
EGFRmu	112 (19)
EGFRwt	466 (81)
Age, y	69 (62-75)
Sex	
Female	323 (56)
Male	255 (44)
Race	
White	523 (90)
Black	39 (7)
Asian	9 (2)
Other	7 (1)
Smoking status	
Never-smoker	111 (19)
Past or current smoker	467 (81)
Pathologic stage	
IA	92 (16)
IB	111 (19)
II	169 (29)
III	206 (36)
Tumor size, cm	2.9 (2.0-4.1)
Mode of surgery	
VATS	269 (46)
Robotic	167 (29)
Open/conversion	142 (25)
Synchronous tumors	
Yes	17 (3)
No	561 (97)

Values are presented as median (interquartile range) or n (%).

EGFRmu, epidermal growth factor receptor mutated lung cancer; EGFRwt, epidermal growth factor receptor wild-type lung cancer; VATS, video-assisted thoracoscopic surgery.

**Table 2:** Comparative Analysis of Demographic and Pathologic Features in Epidermal Growth Factor Receptor Wild-Type vs Mutant Patients

Characteristics	EGFRmu (n = 112)	EGFRwt (n = 466)	*P* Value
Age, y	68 (63-74)	69 (62-75)	.65
Sex			<.001
Female	88 (79)	235 (50)	
Male	24 (21)	231 (50)	
Race			<.001
White	92 (83)	431 (94)	
Black	9 (7)	30 (5)	
Asian	8 (9)	1 (<1)	
Other	3 (1)	4 (1)	
Smoking status			<.001
Never-smoker	55 (49)	56 (12)	
Past smoker or current smoker	57 (51)	410 (88)	
Tumor size, cm	2.6 (1.9-3.6)	3.0 (2.1-4.3)	.01
Pathologic stage			<.001
IA	29 (26)	63 (14)	
IB	29 (26)	82 (18)	
II	27 (24)	142 (30)	
III	27 (24)	179 (38)	
Metastatic recurrence			.28
Yes	37 (33)	182 (39)	
No	75 (67)	284 (61)	
Brain metastasis (among recurrent cases)			.18
Yes	16 (43)	56 (31)	
No	21 (57)	126 (69)	
Mode of surgery			.08
VATS	61 (54)	208 (45)	
Robotic	32 (29)	135 (29)	
Open/conversion	19 (17)	123 (26)	
New Primary			.48
Yes	4 (4)	26 (6)	
No	108 (96)	440 (94)	
Received neoadjuvant chemotherapy			.32
Yes	9 (8)	55 (12)	
No	103 (92)	411 (88)	
Satellite nodules			.69
Yes	7 (6)	36 (8)	
No	105 (94)	430 (92)	
Synchronous tumors			<.001
Yes	12 (11)	5 (1)	
No	100 (89)	461 (99)	

Values are presented as median (interquartile range) or n (%).

EGFRmu, epidermal growth factor receptor mutated lung cancer; EGFRwt, epidermal growth factor receptor wild-type lung cancer; VATS, video-assisted thoracoscopic surgery.

### Survival Analysis

**Figure 1:**
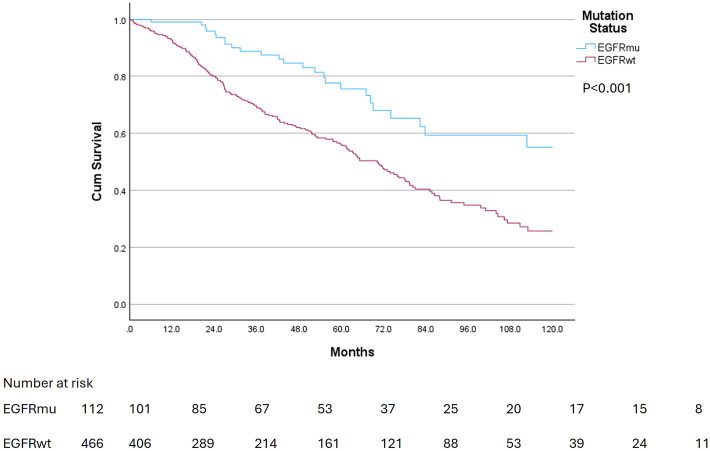
Comparison of 10-year overall survival for stage I-III adenocarcinomas in patients with epidermal growth factor receptor mutated lung cancer (EGFRmu) (blue) and epidermal growth factor receptor wild-type lung cancer (EGFRwt) (red) (*P* < .001).

**Figure 2:**
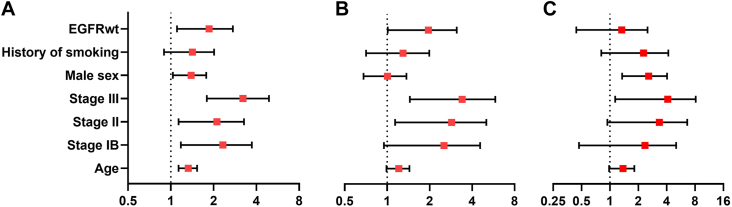
Forest plots showing hazard ratios and 95% CIs for death calculated using multivariable Cox regression. Panels represent risk of death within (A) 10 years, (B) 5 years postrecurrence, and (C) 5 years without disease recurrence. Variables analyzed include epidermal growth factor receptor wild-type status (EGFRwt), smoking history, sex, cancer stage (stage IA reference category), and age.

Mean follow-up time for EGFRmu was 53 months, and 42 months for EGFRwt. Kaplan-Meier analysis showed significant differences in 5-year (75% vs 50%, *P* < .001) and 10-year (55% vs 25%, *P* < .001; Figure 1) overall survival (OS) between EGFRmu and EGFRwt. Stage-specific survival analysis revealed 5-year (95% vs 50%, *P* = .006) and 10-year (75% vs 0%, *P* < .001) survival advantage in only stage IB EGFRmu compared with EGFRwt (Supplemental Figure 2). Multivariable Cox regression analysis identified EGFRwt (HR, 1.74, 95% CI, 1.11-2.74; *P* = .02), male sex (HR, 1.36, 95% CI, 1.03-1.78; *P* = .03), age (HR, 1.32, 95% CI, 1.13-1.53; *P* < .001), stage IB (HR, 2.09, 95% CI, 1.18-3.72; *P* = .01), stage II (HR, 1.93, 95% CI, 1.14-3.28; *P* = .02), and stage-III disease (HR, 2.97, 95% CI, 1.79-4.91; *P* < .001) as independent predictors of worse 10-year OS (Figure 2). The 3-year disease-free survival analysis showed that EGFRmu were less likely to experience disease recurrence within 3 years (75% vs 58%, *P* = .003; Supplemental Figure 3). This difference was statistically significant in stage II (*P* = .046), while a nonsignificant trend favoring EGFRmu was observed in stage IA (*P* = .12; Supplemental Figure 4). Notably, in multivariable analysis, EGFRwt status was not a predictor of death within 5 years when there was no metastatic recurrence (HR, 1.05, 95% CI, 0.44-2.51; *P* = .92), while it remained a significant predictor of death within 5 years after metastatic recurrence occurred (HR, 1.77, 95% CI, 1.01-3.11; *P* = .048; Figure 2).

## Comment

EGFRmu lung cancer presents a unique clinical profile, occurring disproportionately in women and never-smokers. Its unclear etiology makes it a critical area for research, as we currently lack modifiable risk factors and effective screening strategies. Data so far have been inconclusive. Two recent studies have looked at outcomes for patients with EGFRmu who undergo surgery and have found no differences in long-term survival.^4^^,^^5^ On the other hand, a large study conducted in Japan using a cancer registry database found improved survival in EGFRmu patients after adjusting for baseline differences in multivariable regression analysis,^6^ while another study in the US found improved long-term survival in EGFRmu.^7^ These differences are not surprising given the unique demographics that EGFR mutations are associated with, notably female sex, non-smokers, and earlier tumor stage, all of which have historically been linked to improved survival in lung cancer.^8^

Our findings of improved 10-year OS in EGFRmu mirrors the findings of the Japanese registry with similar baseline differences seen between EGFRmu and EGFRwt. When stratified by stage, there were stark survival differences in stage IB and a small trend in stage IA patients that could be explained by the fact that EGFRwt patients include a large subset of NSCLC cases characterized by KRAS mutations. A study by Izar and associates^9^ found KRAS mutations as a prognostic marker for worse disease-free survival and OS in stage I NSCLC compared with KRAS wild-type and EGFRmu, which could explain the differences seen in this study. Interestingly, our multivariable regression analysis revealed that EGFRmu was associated with improved survival only in patients who experienced disease recurrence, with no survival difference observed among those without recurrence. The observed survival benefit may be multifactorial, with one likely contributor being the use of EGFR-TKIs in EGFRmu patients. However, due to the lack of detailed treatment data, this hypothesis remains beyond the scope of the current study. The improved survival is consistent with prior research, which demonstrated significantly improved postrecurrence survival in EGFRmu patients treated with EGFR-TKIs (49 months), compared with EGFRwt or unknown cases (range, 17-20 months) which was due to TKI treatment.^10^

Limitations of our study include its retrospective, single-center design, moderate sample size, use of only lobectomies, lack of information regarding TKI use, and unequal follow-up times between groups. Additionally, the predominance of White and female patients may limit generalizability. Nonetheless, strengths include a wide range of tumor stages and extended follow-up, enabling meaningful comparisons.

EGFR-mutated lung cancer may be a distinct disease from tobacco smoke–induced lung cancer, and the long-term prognosis may be different. Screening and treatment might need to be different for this subset of patients, and additional studies could reveal differences in outcomes.
