# Supplementary material for: Epidermal Growth Factor Receptor Mutation in Resectable Lung Cancer: Association With Survival Outcomes
Source: Ann Thorac Surg Short Rep. 2025 Oct 6;4(1):180–4. doi: 10.1016/j.atssr.2025.09.007 (PMC13100781; doi:10.1016/j.atssr.2025.09.007)
Supplement: Supplementary Figures 1-4 [file mmc1.docx]

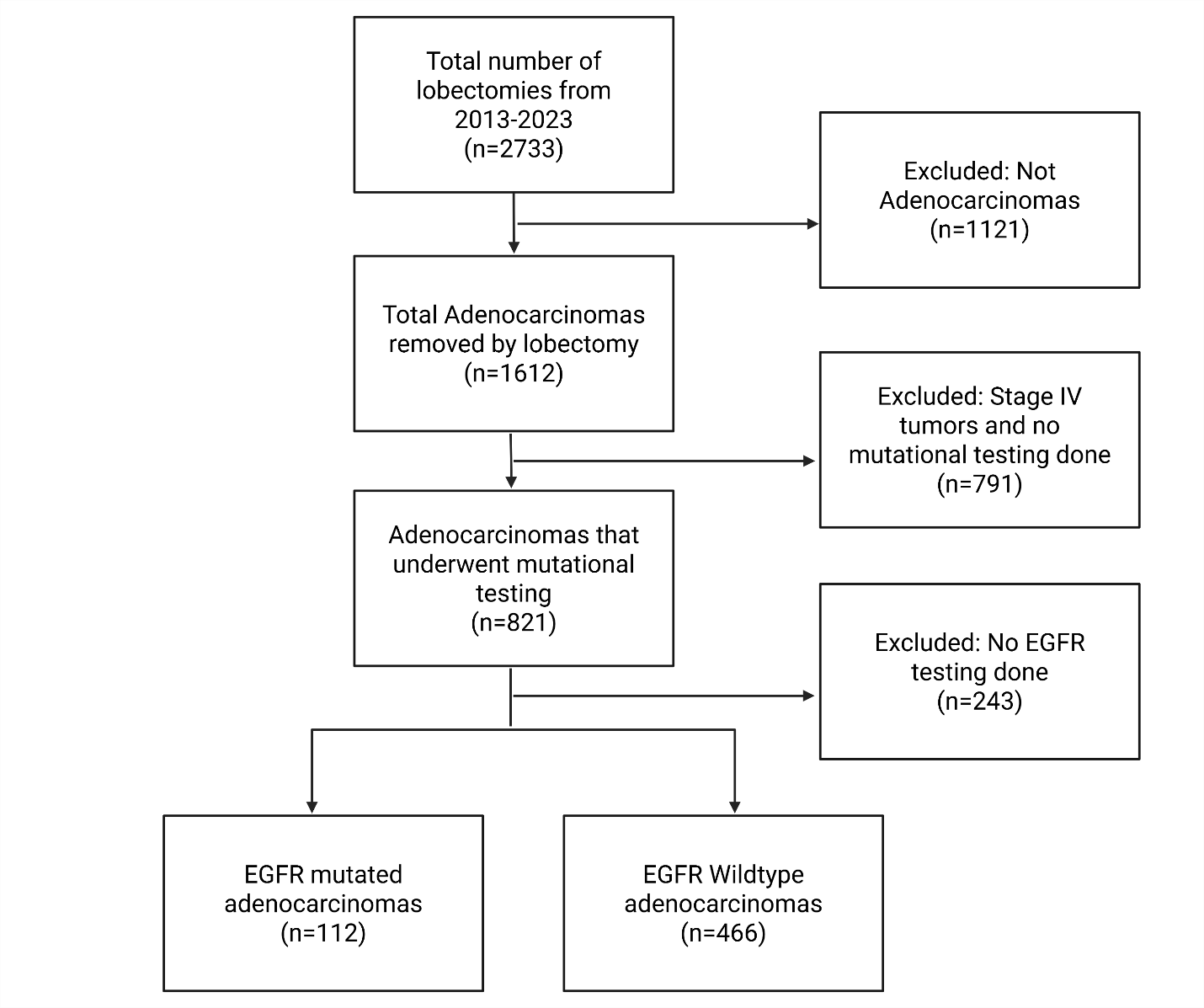


Supplementary figure 1: CONSORT diagram illustrating patient inclusion and exclusion criteria for the study cohort


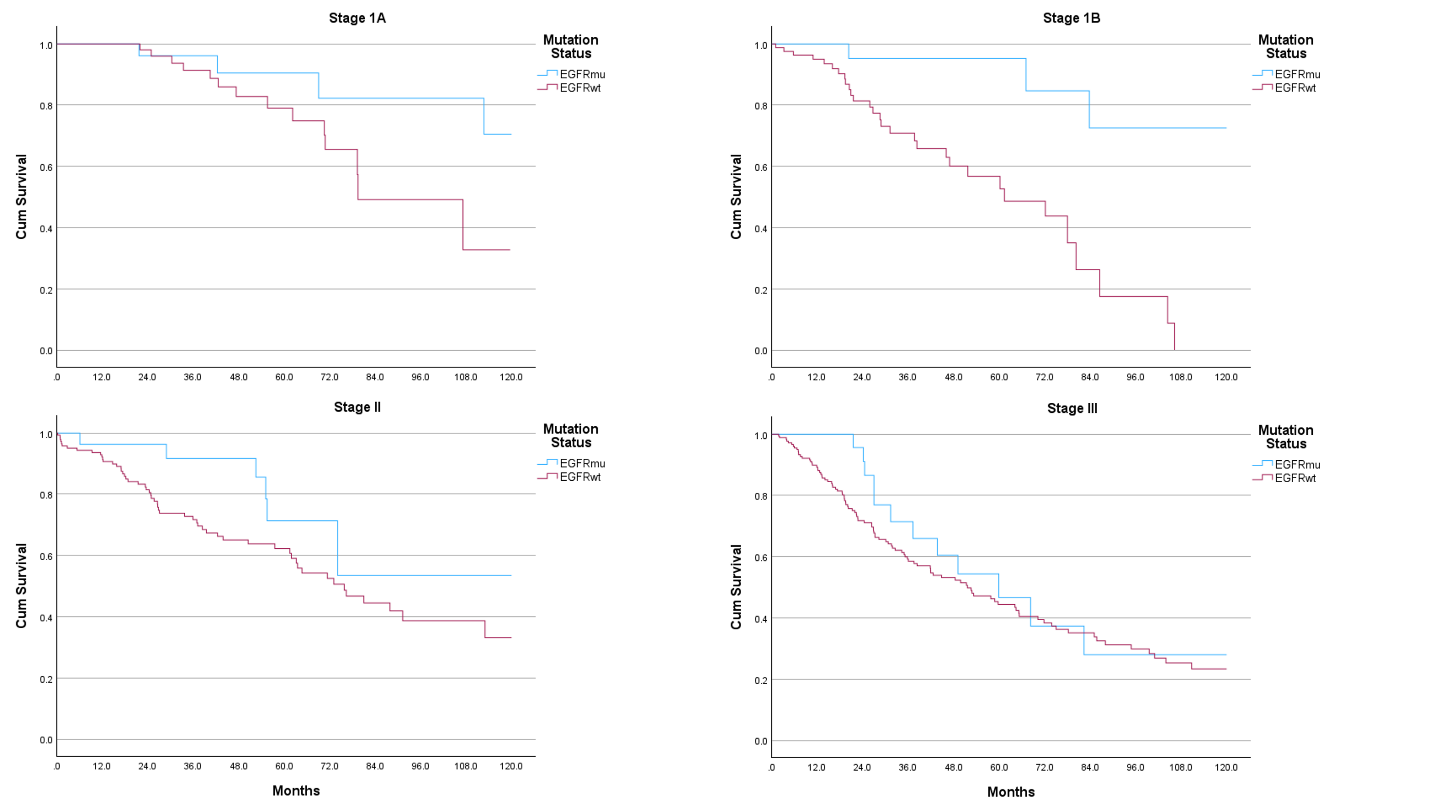


Supplementary figure 2: 10-year overall survival comparison between patients with A) stage IA (p=0.07), B) Stage IB (p<0.001), C) Stage II (p=0.08), and Stage III (p=0.39) EGFR-mutant (EGFRmu) and EGFR wild-type (EGFRwt) lung cancer


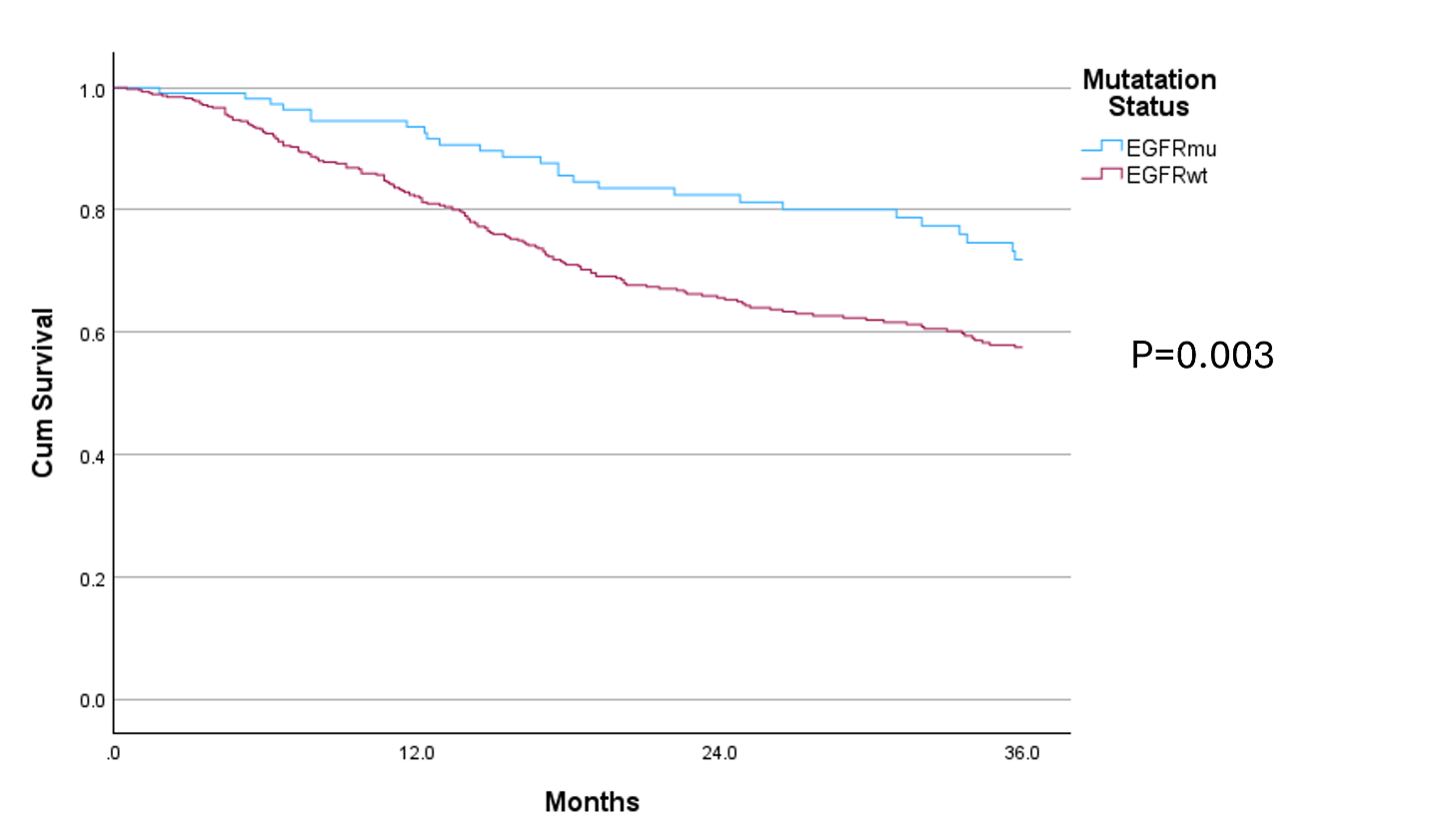


Supplementary figure 3: Three-year disease-free survival comparison between patients with stage I–III EGFR-mutant (EGFRmu) and EGFR wild-type (EGFRwt) lung cancer


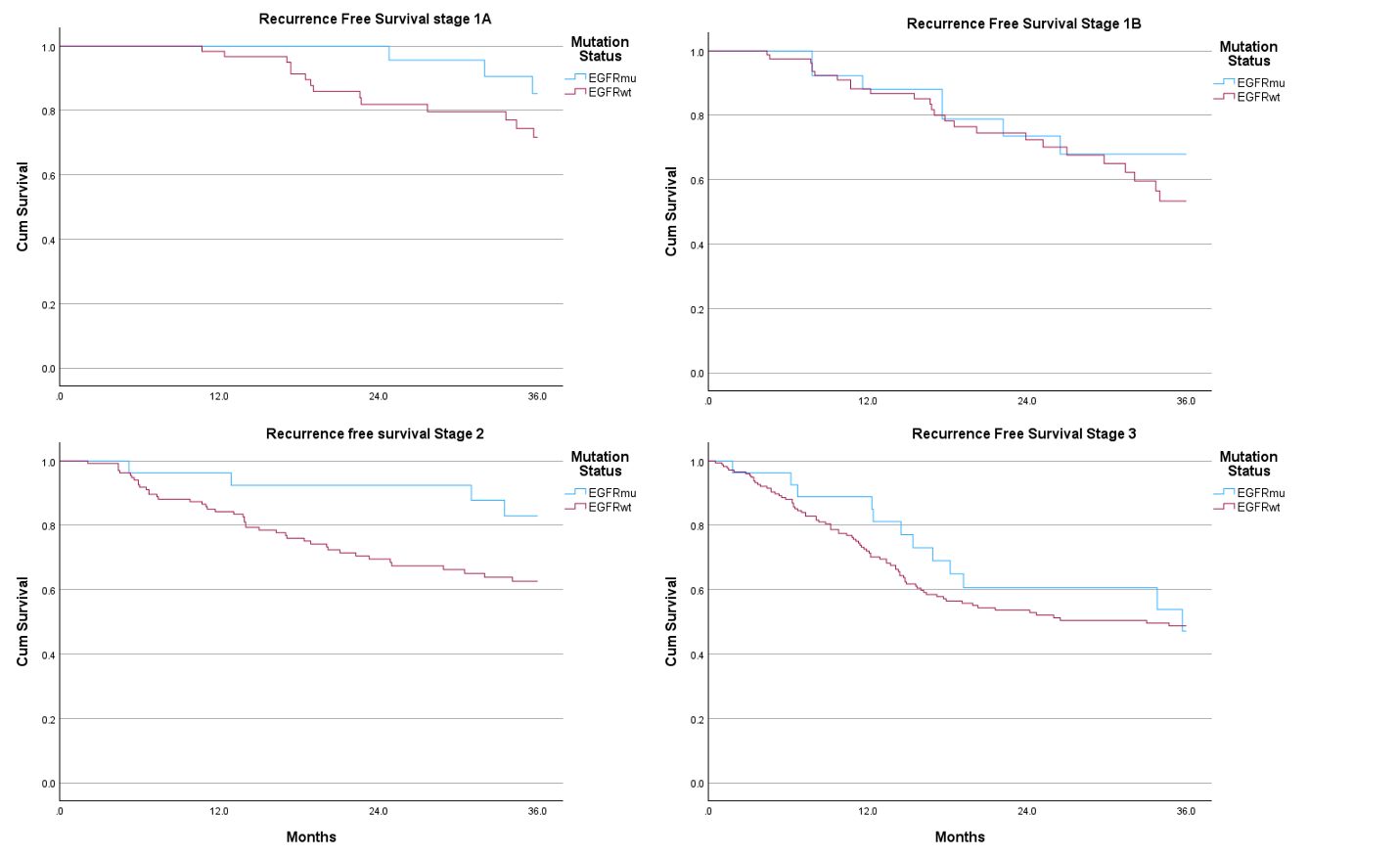


Supplementary figure 4: Three-year disease-free survival comparison between patients with stage IA (p=0.12), Stage IB (p=0.39), Stage II (p=0.046), and Stage III (p=0.55) EGFR-mutant (EGFRmu) and EGFR wild-type (EGFRwt) lung cancer

Supplementary figure 1: CONSORT diagram illustrating patient inclusion and exclusion criteria for the study cohort
